# Effect of sub-technique transitions on energy expenditure and physiological load in the classical-style technique among elite male cross-country skiers

**DOI:** 10.1007/s00421-021-04783-5

**Published:** 2021-08-17

**Authors:** Tomas Carlsson, Lars Wedholm, Wilma Fjordell, Mikael Swarén, Magnus Carlsson

**Affiliations:** 1 School of Education, Health and Social Studies, Dalarna University, Högskolegatan 2, 791 88 Falun, Sweden; 2 Swedish Unit for Metrology in Sports, Dalarna University, Högskolegatan 2, 791 88 Falun, Sweden

**Keywords:** Cross-country skiing, Diagonal-stride technique, Double-poling technique, Metabolic stress, Gait transition, Blood lactate concentration

## Abstract

**Purpose:**

To investigate whether sub-technique transitions in the classical-style technique are associated with increased energy expenditure and/or metabolic stress among elite male cross-country skiers.

**Methods:**

Fifteen elite male skiers completed three 10-min treadmill roller-skiing tests, each of which consisted of 5 min using the diagonal-stride technique (DS) and 5 min using the double-poling technique (DP), combined in three various modes all ensuring comparable mechanical workload, at an inclination of 2.5° and a speed of 13 km/h. In the first and third tests, the participants used 5 min continuous DS followed by 5 min continuous DP, or vice versa (no transition (NT) test), whereas in the second test, they made transitions between DS and DP every 6 s (repeated transition (RT) test). The last 3 min of each 5-min stage was used to calculate the mean values of oxygen uptake (${\dot{\text{V}}\text{O}}_{{2}}$), respiratory exchange ratio (RER), metabolic rate (MR), mechanical work rate (MWR), and gross efficiency (GE). In addition, the pre–post-difference in blood lactate concentration (La_diff_) was determined for each test. Paired-samples *t* tests were used to investigate differences between tests.

**Results:**

There were no significant differences between NT and RT tests regarding V̇O_2_, MR, MWR, or GE. Conversely, significant differences were found in RER and La_diff_, where the NT test was associated with higher RER and La_diff_ values.

**Conclusions:**

Roller skiing with repeated sub-technique transitions is not associated with an additional aerobic energy contribution; instead, the anaerobic energy contribution was lower compared to that under continuous use of DS and DP.

## Introduction

Currently, competitions in cross-country skiing are performed using classical technique; free technique; or a skiathlon format, where the first and second halves of the race are conducted using classical technique and free technique, respectively. All the competitions should, according to the International Ski Federation’s (FIS) regulations, be conducted on homologated courses with equal proportions of uphill sections, sections with undulating terrain, and downhill sections (FIS 2018). As a consequence of the varying terrain characteristics and skiing speeds during a race in classical or free technique, the skiers must repeatedly change sub-techniques within the technique.

In the classical technique, there are four main sub-techniques, known as ‘gears’, that are used to produce propulsive force (Nilsson et al. 2004): the double-poling technique (DP), the double poling with leg kick technique (DPK), the diagonal-stride technique (DS), and the herringbone technique. In downhill sections, the non-propulsive tuck technique is used to minimise air resistance, thereby increasing or maintaining skiing speed or minimising its reduction. These sub-techniques are alternated depending mainly on the inclination of the actual course section (Ettema et al. 2017; Løkkeborg and Ettema 2020; Pellegrini et al. 2013). In general, DP is used on slight downhill, flat and slight uphill sections where the skiing speed is relatively high, whereas a transition to DPK is made when course inclination increases. However, the use of DPK in competitions is currently relatively low (Marsland et al. 2017), and elite skiers change instead ‘gear’ directly from DP to DS when entering uphill sections with moderate or steep inclines where the skiing speed decreases. The sub-technique transition between DP and DS or vice versa has recently been shown to be the most frequently used transition between sub-techniques with propulsive force production (Solli et al. 2018).

Analyses of movement patterns during a 10-km competition (2 laps around an ~ 5.5 km loop) on an FIS-homologated course showed that DP was the most frequently used sub-technique (~ 41% of the time), followed by DS (~ 24%), the tuck technique (~ 16%), DPK (~ 7%), and the turning technique (~ 5%) (Marsland et al. 2017). The remaining 7% of the race time was attributed to 279 ± 18 sub-technique transitions per skier (mean ± standard deviation) during the competition. Hence, sub-technique transitions account for a sizeable proportion of the total race time; therefore, the timing and efficiency of the transitions could be important factors for performance.

Previously, it was shown that the course inclination where skiers make transitions between different sub-techniques in the classical technique varies somewhat between individuals (Pellegrini et al. 2013). This result is supported by results in a recent study (Stöggl et al. 2018), which found individual differences in sub-technique usage on uphill sections with a mean inclination of 3.5° during distance races in classical technique; faster skiers used less DS and more DPK than slower skiers, whereas the usage of DP was equal for the two categories of skiers. They also reported that women performed more sub-technique transitions than men (Stöggl et al. 2018), which together indicate that the skier’s physiological capacity is an important factor affecting when and how often sub-technique transitions are employed.

The triggering mechanisms of human gait transitions have been thoroughly investigated for the transition between walking and running, and these gait transitions are reported to occur over a few steps to maintain balance between these two modes of exercise (Hagio et al. 2015; Segers et al. 2013). In a recently published review, four main mechanisms have been suggested to trigger gait transitions (Kung et al. 2018): metabolic efficiency, mechanical efficiency, mechanical loading, and cognitive and perceptual triggers. The ultimate goal with gait transitions is to minimise the metabolic cost of movement (Minetti et al. 1994); however, changes in energy expenditure are a relatively slow process, which indicates that metabolic efficiency is not the most important trigger mechanism because rapid feedback is not available (Kung et al. 2018). The other three trigger mechanisms offer a fast response and could thereby initiate transitions between exercise modes when a specific threshold value is reached. The mechanical-efficiency trigger is believed to initiate the gait transition when movement becomes less efficient from a mechanical perspective (e.g. centre-of-mass displacement) to decrease the mechanical cost (Farley and Ferris 1998; Minetti et al. 1994). The trigger related to mechanical load is suggested to reduce the stress on the musculoskeletal system by acting as an injury-protective mechanism (Hreljac 1993; Raynor et al. 2002). Furthermore, cognitive processes and perceptual feedback are proposed to influence the timing of the transition (Kung et al. 2018). As previously mentioned, the overall purpose of these trigger mechanisms is to minimise the metabolic cost using an energy-efficient movement pattern.

In cross-country skiing, pole-force and leg-thrust-time thresholds have been reported to trigger sub-technique transitions in the classical technique (Pellegrini et al. 2013); these transitions are related to mechanical-load and mechanical-efficiency triggers. From a metabolic-efficiency perspective, it was previously shown that elite skiers execute the transition from DP to DS at a metabolic-cost equilibrium, but the DS-to-DP transition was performed when the energetic cost of DP was considerably higher than that of DS (Andersson et al. 2016). In a recently published systematic review, it was reported that performance in cross-country skiing appears to be improved by joint, whole-body, ski, and pole kinematics that promote forward propulsion while minimising unnecessary movement (Zoppirolli et al. 2020). Hence, skiers make necessary sub-technique transitions to adapt to the actual speed and inclination to increase mechanical efficiency (Dahl et al. 2017; Løkkeborg and Ettema 2020), which contributes to a reduction in energy expenditure. From this perspective, the skier should be responsive to feedback from the trigger mechanisms signalling when to make sub-technique transitions.

It has been suggested that the ability to make energy-efficient sub-technique transitions is an important factor for cross-country skiing performance (Losnegard 2019; Sandbakk and Holmberg 2014), which implies that sub-technique transitions are associated with an additional energy cost. However, to the best of our knowledge, no previous research study has investigated the effect of sub-technique transitions in cross-country skiing in terms of energy cost. The purpose of this study was to investigate whether sub-technique transitions in the classical-style technique are associated with increased energy expenditure and/or metabolic stress among elite male cross-country skiers.

## Methods

### Participants

Fifteen elite male cross-country skiers (age 22 ± 4 years; stature 183 ± 9 cm; body mass 79.0 ± 9.9 kg) volunteered to participate in the study. Ten of the skiers had competed in the World Ski Championships and/or the World Cup. All subjects gave their written informed consent to participate in the study. The test procedures were performed in accordance with the World Medical Association’s Declaration of Helsinki—Ethical Principles for Medical Research Involving Human Subjects 2008, and the study was approved by the Swedish Ethical Review Authority, Lund, Sweden (Dnr 2020-00775).

### Testing procedures

The participants were instructed to perform only light training on the 2 days preceding their scheduled test days, to be well hydrated, to refrain from alcohol (24 h) and caffeine (12 h) and to avoid eating within 2 h prior to testing. On the day of the tests, the participants completed a health-status questionnaire, and thereafter, each participant’s stature (Harpenden Stadiometer, Holtain Limited, Crymych, Great Britain) and body mass (Midrics 2, Sartorius AG, Goettingen, Germany) were measured.

The three roller-skiing tests included in the study were performed on a motor-driven treadmill (Saturn, 450/300rs, h/p/cosmos sports & medical GmbH, Nussdorf-Traunstein, Germany), and throughout the tests, the participants used their own poles along with plastic tips (black plastic tip; LEKI Lenhart GmbH, Kirchheim, Germany) and roller skis (Pro-Ski C2, Sterner Specialfabrik AB, Dala-Järna, Sweden) provided by the laboratory. The coefficient of rolling resistance of the roller skis (*μ*) was determined to be 0.022 using the negative-inclination-equilibrium method previously described (Carlsson et al. 2016). The tests were video recorded (HC-V750, Panasonic, Osaka, Japan) to allow video analyses of diagonal-stride and double-poling frequencies during the tests with Dartfish Live S, Version 10 (Dartfish SA, Fribourg, Switzerland).

Prior to the tests, the participants performed a standardised familiarisation/warm-up treadmill roller skiing. During a 7-min period, the participants were familiarised with treadmill inclination and speed in the test protocol as well as the sub-technique transition procedure with a change between DS and DP every 6 s. Thereafter, the participants completed a 12-min warm-up consisting of the diagonal-stride technique (~ 10 km/h, 5°) and double-poling technique (~ 13 km/h, 2.5°). The warm-up was followed by 27 min of rest before the first test was initiated. During the rest before each test, each participant ate 1/3 of a banana (~ 50 g) and was allowed to drink water to maintain the blood-glucose concentration and fluid balance.

Throughout the roller-skiing tests, expired air was continuously analysed using a metabolic cart in mixing-chamber mode (Jaeger Oxycon Pro, Erich Jaeger Gmbh, Hoechberg, Germany). The metabolic cart was calibrated according to the specifications of the manufacturer before each test, and before each new test, a ‘zeroing’ of the O_2_ and CO_2_ sensors was performed.

One minute before the 3-min pre-phase of each test and 1 min after completion of the 10-min test, capillary blood samples were collected from a fingertip and thereafter analysed to determine blood lactate concentrations (Biosen 5140, EKF-diagnostic GmbH, Barleben, Germany).

### Roller-skiing tests

**Fig. 1:**
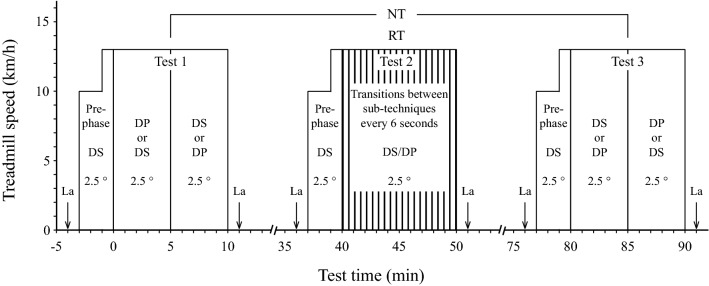
Overview of the test procedure, where La is blood lactate sampling, DS is diagonal-stride technique, DP is double-poling technique, NT is the roller-skiing tests with no sub-technique transitions (Test 1 + Test 3), and RT is the roller-skiing test with repeated sub-technique transitions (Test 2)

To determine the energy cost and metabolic stress related to sub-technique transitions in classical-technique cross-country skiing, three treadmill roller-skiing tests were performed separated by 27 min of rest (Fig. 1). Before each new test, the total mass (*m*_e_) of the participant and his equipment (i.e. roller skis, poles, ski boots, safety harness, heart-rate receiver, gloves, and clothes) was measured to be able to calculate gross efficiency (GE). All three tests consisted of a 3-min pre-phase where the participants skied for 2 min at a treadmill speed (*v*) of 10 km/h and a treadmill inclination (*α*) of 2.5° using DS. During the last minute of the pre-phase, the treadmill speed was increased to 13 km/h while maintaining an inclination of 2.5°, which corresponded to the speed/inclination combination that was used in all three tests. This combination was chosen based on the following criteria: (1) the speed and inclination should be suitable for roller skiing using both DS and DP, (2) all participants should have a mean respiratory exchange ratio (RER) below 1.0 to enable calculation of GE, and (3) the participants’ baseline value of metabolic stress (i.e. blood lactate concentration) before each new test should not differ between tests.

Directly after the end of the 3-min pre-phase, the 10-min test was initiated while maintaining a treadmill speed of 13 km/h and a treadmill inclination of 2.5°. A counterbalanced test design was applied, where eight of the participants were randomly assigned to start with 5 min of roller skiing using DS, which was followed by 5 min of roller skiing using DP. The other seven participants performed the sub-techniques in the opposite order during the first test.

During the second test, which contained repeated transitions between sub-techniques (RT test), the participants made sub-technique transitions every 6 s (i.e. 10 transitions per minute). The time between transitions (i.e. number of transitions during the test) was selected, based on a pilot study, to be able to detect a potential low energy cost associated with sub-technique transitions, without influence the skiing between transitions negatively. Each transition was preceded by a 3-s countdown (Stopwatch, Fitlb, San Jose, USA) with one beep per second followed by a higher tone that was regarded as the intended time of the sub-technique transition.

During the third test, the sub-technique order was alternated compared to the first test; hence, the eight participants who started with DS in the first test now started with 5 min using DP followed by 5 min using DS. The other seven participants performed the sub-techniques in the opposite order.

Each of the three tests contained 10 min of roller skiing, comprising 5 min of each sub-technique. Data for the subsequent calculations and statistical analyses were collected during the last 3 min of each 5-min stage (i.e. from minutes 2 to 5 and from minutes 7 to 10). For each test, these two 3-min values were used to calculate a mean value for each test variable. To control for potential physiological drift and test order, the data from tests 1 and 3 were combined to represent the test condition with no sub-technique transitions (NT test). Hence, to quantify the energy expenditure and metabolic stress associated with the sub-technique transitions, the test variables from the NT and RT tests were compared.

The calculations of metabolic rate (MR) and GE were based on the mean oxygen uptake (${\dot{\text{V}}\text{O}}_{{2}}$) (l/min) and RER (l/l) using the equations presented previously (Carlsson et al. 2016). The MR (J/s) was determined using the formula (3.815 + 1.232 · RER) · ${\dot{\text{V}}\text{O}}_{{2}}$ · *k*_1_, where *k*_1_ is 0.251 and converts kcal/min to J/s (i.e. W). The GE is the ratio of the mechanical work rate (MWR) to MR. The MWR (J/s) is the sum of the work against gravity and the work related to overcoming the rolling resistance of the roller skis; the MWR was calculated in accordance with the formula: (*m*_e_ · *g* · sin *α* · *v* + *k*_2_ · *m*_e_ · *g* · cos *α* · *μ* · *v*), where *g* is the acceleration due to gravity; *k*_2_, the ratio of the average force applied to the roller skis during the forward rolling phase to the force caused by body mass and gravity, is set to 0.53 for DS (Carlsson et al. 2012) and 1.00 for DP (Carlsson et al. 2017).

### Statistical analyses

Test results are presented as the means and standard deviations. The normality of the distributions of physiological variables was assessed using the Shapiro–Wilk test. To determine the effect of sub-technique transitions on energy expenditure and metabolic stress in the classical-style technique, paired-samples Student’s *t* tests (*t*) were used to investigate test-variable differences between NT and RT tests. Cohen’s effect-size criteria were used to interpret the magnitude of the effect size (*η*^2^) and to enable more informative inferences to be made from the results. The substantial effects were divided into more finely graded magnitude ranges as follows: small effect for 0.01 ≤ *η*^2^ < 0.06, moderate effect for 0.06 ≤ *η*^2^ < 0.14, and large effect for *η*^2^ ≥ 0.14 (Cohen 1988). One-way repeated measures analysis of variance (ANOVA) was used to compare the pre–post-blood lactate difference (La_diff_) between the tests. All statistical analyses were assumed to be significant at an alpha level of 0.05. The statistical analyses were conducted using IBM SPSS Statistics software, Version 26 (IBM Corporation, Armonk, USA).

## Results

The test results for the NT and RT tests are presented in Table 1. Significant differences between NT test and RT test were found for RER and La_diff_ (Table 1); however, there were no significant differences between tests for the variables ${\dot{\text{V}}\text{O}}_{{2}}$, MR, MWR, and GE (Table 1).

**Table 1:** Results from the roller-skiing tests

Variable	NT	RT	NT-RT diff	*t*	*P*	*η*^2^
${\dot{\text{V}}\text{O}}_{{2}}$ (l/min)	3.56 ± 0.40	3.57 ± 0.40	− 0.01 ± 0.05	− 0.88	0.39	0.052
RER (l/l)	0.90 ± 0.03	0.88 ± 0.02	0.02 ± 0.01	5.77	< 0.001	0.70
MR (J/s)	1221 ± 137	1221 ± 138	0 ± 1	0.16	0.88	0.002
MWR (J/s)	176 ± 21	176 ± 21	0 ± 0	0.84	0.42	0.048
GE (%)	14.4 ± 0.6	14.4 ± 0.5	0.0 ± 0.2	− 0.11	0.91	0.001
La_diff_ (mmol/l)	0.41 ± 0.61	− 0.11 ± 0.54	0.52 ± 0.77	2.64	0.019	0.33

All test values are presented as the mean ± standard deviation. *NT* mean results from the two roller-skiing tests that include 5 min using the double-poling technique and 5 min using the diagonal-stride technique, *RT* the 10-min roller-skiing test with repeated sub-technique transitions where the technique was changed every 6 s, *NT-RT diff* the paired difference between NT and RT test, $\dot{V}O_{2}$ mean oxygen uptake, *RER* mean respiratory exchange ratio, *MR* metabolic rate, *MWR* mechanical work rate, *GE* gross efficiency, *La*_*diff*_ difference in blood lactate concentration between post-test and pre-test. Differences between NT and RT were investigated using paired-samples *t* test where *P* values and effect sizes (*η*^2^) are presented

The blood lactate concentrations before and after the RT test were 1.08 ± 0.30 mmol/l and 0.97 ± 0.51 mmol/l, respectively. The corresponding blood lactate concentrations for the NT test were 1.17 ± 0.27 mmol/l (pre) and 1.58 ± 0.59 mmol/l (post). The 10-min tests included in the NT test that ended with 5 min using either DP or DS had La_diff_ of 0.68 ± 0.67 mmol/l (pre, 1.13 ± 0.38 mmol/l; post, 1.81 ± 0.74 mmol/l) and 0.15 ± 0.67 mmol/l (pre, 1.13 ± 0.48 mmol/l; post, 1.31 ± 0.50 mmol/l), respectively. The ANOVA revealed that there is a significant difference in La_diff_ (*F*_2,28_ = 8.08; *P* = 0.0017; *η*^2^ = 0.37), and the post hoc tests showed a significantly greater La_diff_ for the test that ended with DP compared with the RT test (*t* = 3.61; *P* = 0.0028; *η*^2^ = 0.48) and the test that ended with DS (*t* = 3.13; *P* = 0.0074; *η*^2^ = 0.41). No significant difference in La_diff_ was found between the RT test and the test that ended with DS (*t* = − 1.26; *P* = 0.22; *η*^2^ = 0.10). The technique-specific GE during the NT test was 16.9 ± 0.6% for DP and 12.3 ± 0.6% for DS, with a significantly higher GE for DP (*t* = 46.6; *P* < 0.001; *η*^2^ = 0.99).

The diagonal-stride frequency was 0.75 ± 0.05 Hz in the NT test and 0.72 ± 0.04 Hz in the RT test, and a significant difference was found between tests (*t* = 4.08; *P* = 0.0011; *η*^2^ = 0.54). The double-poling frequencies were 0.86 ± 0.06 Hz and 0.88 ± 0.06 Hz for the NT test and RT test, respectively; no significant difference in poling frequencies was found between the tests (*t* = − 1.82; *P* = 0.091; *η*^2^ = 0.19).

## Discussion

The results of this study demonstrate that sub-technique transitions in classical-style cross-country skiing are not related to an increased energy expenditure compared to skiing with no transitions. Moreover, as indicated by significantly increased blood lactate concentrations after the test with no transitions, the anaerobic energy contribution appears to be reduced during the test with repeated sub-technique transitions.

The method used in the current study was designed to have equal mean MWR in the NT and RT tests (Table 1). The difference between the NT and RT tests was the number of transitions; the RT test contained 60 sub-technique transitions during the 6 min that were included in the analysis, whereas the NT test contained no sub-technique transitions in the corresponding time period. The results show no significant difference in ${\dot{\text{V}}\text{O}}_{{2}}$ between the NT and RT tests, despite the frequent sub-technique transitions in the RT test. Hence, the aerobic energy contribution to the work performed did not differ between the two test conditions. From a physiological perspective, this is somewhat expected because the musculature involved in power production in each technique has to perform an equal amount of work independent of whether the work is performed using frequent sub-technique transitions between DP and DS or using DP and DS continuously for 5 min each. From a physics perspective, it could be speculated that the transition between sub-techniques results in a greater reduction in skiing speed from one muscle-force transfer to the next compared to speed reductions between two stride cycles within the same sub-technique; this would potentially increase the power demand related to translational kinetic energy when the skier makes sub-technique transitions (Bergh 1987). Moreover, during the stride cycle, a number of body segments rotate in relation to their joints, and the power demand is related to each segment’s moment of inertia and the square of its angular velocity (Bergh 1987).

A changed movement pattern during the transition between sub-techniques might cause an increased power demand related to rotational kinetic energy, which in turn would lead to additional energy expenditure during the RT test. However, despite some mistimed transitions caused by ‘bad timing’ of the intended time of the sub-technique transition, there was no significant difference in either MR or GE between the tests. Hence, it seems that elite skiers have the ability to make transitions between sub-techniques without reducing their skiing speed more than they would between two movement cycles within a sub-technique at a submaximal work intensity. Another possible explanation for the non-significant MR difference between tests could be elevated energy expenditure during the NT test that outbalances a possible increase in MR related to translational and rotational kinetic energy differences between the tests.

When comparing DP and DS during uphill skiing, the muscle-activity pattern differs between techniques (Holmberg et al. 2005; Vähäsöyrinki et al. 2008). The primary working muscles in DP during the poling phase are the muscles in the upper body, whereas the lower body musculature is more active during the recovery phase of the DP cycle (Bojsen-Møller et al. 2010; Holmberg et al. 2005). Recently, it was shown that approximately 40% of the contribution to power production during ergometer DP is derived from the lower body at a moderate work intensity (Danielsen et al. 2018), close to the intensity used in the current study; when exercise intensity increases, the lower body muscles contribute increasingly (Bojsen-Møller et al. 2010; Danielsen et al. 2018; Rud et al. 2014).

Previously, it was reported that the arm’s muscle activity (% of maximal voluntary contraction) during the poling phase is significantly higher during DP than during DS at the same relative submaximal work intensity; this difference is accompanied by approximately threefold higher peak pole forces during DP (Björklund et al. 2015). Moreover, a pole-force ratio of three between DP and DS was also reported in a study where a treadmill speed and inclination of 12.5 km/h and 2.3° were used, respectively, which is a speed/inclination combination that is almost identical to that used in the current study (Dahl et al. 2017). Force production by the arms during DP, even at moderate intensities, is suggested to lead to mechanical hindrance of the oxygen supply, resulting in a lower oxygen extraction in the arms than in the legs (Stöggl et al. 2013). This oxygen-extraction difference between the arms and legs is explained by factors such as a higher heterogeneity in blood-flow distribution, smaller diffusing area, larger diffusing distance, and shorter mean transit time in the arms (Calbet et al. 2005). The impaired oxygen supply to the arm muscles implies that there is higher reliance on glycolytic type II muscle fibres for force production and thereby increased lactate production (Ahlborg and Jensen-Urstad 1991; van Hall 2000). Accordingly, there is a positive lactate arteriovenous difference in the arms during DS (Björklund et al. 2010) and DP (Stöggl et al. 2013), indicating that there is a net increase in blood lactate from the arms during submaximal treadmill roller skiing. Together, these findings could explain the significantly greater La_diff_ for the test that ended with 5 min using DP compared to the test that ended with 5 min using DS, which both were included in the NT test. Hence, the recovery time during the double-poling cycle appears to be too short to avoid an elevated blood lactate concentration, which is supported by the previous observation of a longer recovery phase during DP related to a reduced blood lactate concentration at a given submaximal work intensity (Holmberg et al. 2006).

The significantly higher La_diff_ and RER in the NT test indicate that the anaerobic energy contribution is greater than that in the RT test. Consequently, the total energy contribution during the RT test is, in fact, not greater than that during the NT test, despite repeated sub-technique transitions. If the actual transition between sub-techniques is related to an energy cost, then this potential energy cost is outbalanced by the increased anaerobic energy contribution during the NT test. The reason for the lower La_diff_ during the RT test, despite having the same amount of roller skiing in each technique, is most likely attributed to the frequent transitions between sub-techniques. By alternating DP and DS every 6 s, the load on the arms is reduced while using DS, which entails a lower mechanical hindrance of the blood flow and thereby a better oxygen supply and lactate clearance. As a consequence of better oxygenation of the arm musculature when a new bout of DP is initiated, the amount of anaerobic energy contributed by glycolytic processes is reduced, and a greater relative proportion of the energy contribution for propulsion comes from aerobic processes. This plausible explanation is supported by the negative La_diff_ in the RT test. Hence, the novel finding in the current study that skiing with frequent sub-technique transitions is not related to an increased energy cost compared to that under continuous use of DP and DS could be attributed to either no additional energy cost for the transition between sub-techniques or a condition where a potential energy cost for the transitions is outbalanced by an elevated energy expenditure during the NT test.

Previously, the cost of locomotion for national-level cross-country skiers was found to be equal for DP and DS at a fixed speed of 10 km/h when the treadmill inclination was approximately 2.5° (Pellegrini et al. 2013). However, in the current study, there was a significant difference in GE between DP and DS, where DP was associated with a lower MR. This finding is supported by a recent study that found a significantly higher GE for DP than DS at a 2.9° incline (Dahl et al. 2017). The difference in steady-state oxygen consumption between techniques during the NT test, reflected by the GE difference, indicates that the difference is too large to be able to adapt to within 6-s bouts during the RT test. Therefore, the ${\dot{\text{V}}\text{O}}_{{2}}$ kinetics might influence the MR during the RT test. Future studies within this area should use an inclination where the GE is as equal as possible between the sub-techniques. Because GE has been shown to be higher for DS than DP at an inclination of 5° (Andersson et al. 2021), the GE equilibrium should be somewhere between a 2.9° and 5° incline for elite male skiers.

Another limitation of the current study is that the work intensity of the task differs from the intensity of actual cross-country skiing races. However, when the work intensity increases from submaximal to maximal, there are several force-related characteristics that change. For example, the pre-activation time of the force-producing musculature increases, the rate of force development increases, the peak pole and leg-kick forces increase, and the force-application time decreases in both DS (Andersson et al. 2014; Björklund et al. 2010; Vähäsöyrinki et al. 2008) and DP (Lindinger et al. 2009; Stöggl and Holmberg 2016; Stöggl and Müller 2009). The enhanced force production is reflected by an elevated MR when the work intensity increases. A potential energy cost for the transition between sub-techniques will then be ‘diluted’ in the elevated MR, which, in turn, makes it reasonable to assume that the influence of sub-technique transitions on the total MR is low. Based on this reasoning and the results of the current study, sub-technique transitions should not substantially influence skiing performance. Therefore, the general recommendation in a recent study that skiers should make fewer sub-technique transitions on intermediate terrain (2°–5°) to improve performance in distance races in the classical technique (Stöggl et al. 2018) could be questioned, especially since this conclusion is drawn based on sub-technique differences between groups of fast and slow skiers.

The findings in the current study indicate that a pre-determined strategy with frequent shifts between sub-techniques could be appropriate from the perspective of minimising the metabolic load. This strategy could be applied on course sections where at least two sub-techniques are feasible in terms of course inclination, because inclination have been proposed to be main factor shifts in technique (Ettema et al. 2017; 2018). Future studies are needed to further investigate whether a strategy with frequent sub-technique transitions could reduce the metabolic stress in elite cross-country skiers at the work intensity of a real competition or for skiers with different technical abilities.

## Conclusions

Roller skiing with repeated sub-technique transitions in classical-style skiing is not associated with an additional aerobic energy contribution compared to that under continuous use of DS and DP among elite male cross-country skiers. Instead, roller skiing with repeated sub-technique transitions is associated with reduced metabolic stress/anaerobic energy contribution (i.e. a lower blood lactate concentration).

## Abbreviations

*α*: Treadmill inclination
DP: Double-poling technique
DPK: Double-poling with leg kick technique
DS: Diagonal-stride technique
*η*^2^: Effect size
FIS: International Ski Federation
*g*: Gravitational acceleration
GE: Gross efficiency
La: Blood lactate concentration
*m*_e_: Total mass of participant and equipment
MR: Metabolic rate
MWR: Mechanical work rate
NT: No transitions between sub-techniques
RER: Mean respiratory exchange ratio
RT: Repeated transitions between sub-techniques
*t*: *t*-value from a paired-samples Student’s *t* test
*μ*: Coefficient of rolling resistance of the roller skis
*v*: Treadmill speed
${\dot{\text{V}}}$O_2_: Mean oxygen uptake
